# PPAR*δ* Activity in Cardiovascular Diseases: A Potential Pharmacological Target

**DOI:** 10.1155/2009/745821

**Published:** 2009-03-23

**Authors:** Angela Tesse, Ramaroson Andriantsitohaina, Thierry Ragot

**Affiliations:** ^1^Laboratoire de Biologie Neurovasculaire Intégrée (LBNVI), UMR CNRS 6214/INSERM 771, Faculté de Médecine, Université d'Angers, rue Haute de Reculée, 49045 Angers Cedex 01, France; ^2^Institut de Cancérologie Gustave Roussy (IGR), CNRS UMRY 8121, PR2, 39 rue Camille Desmoulins, 94805 Villejuif, France

## Abstract

Activation of peroxisome proliferator-activated receptors (PPARs), and particularly of PPAR*α* and PPAR*γ*, using selective agonists, is currently used in the treatment of metabolic diseases such as hypertriglyceridemia and type 2 diabetes mellitus. PPAR*α* and PPAR*γ* anti-inflammatory, antiproliferative and antiangiogenic properties in cardiovascular cells were extensively clarified in a variety of in vitro and in vivo models. In contrast, the role of PPAR*δ* in cardiovascular system is poorly understood. Prostacyclin, the predominant prostanoid released by vascular cells, is a putative endogenous agonist for PPAR*δ*, but only recently PPAR*δ* selective synthetic agonists were found, improving studies about the physiological and pathophysiological roles of PPAR*δ* activation. Recent reports suggest that the PPAR*δ* activation may play a pivotal role to regulate inflammation, apoptosis, and cell proliferation, suggesting that this transcriptional factor could become an interesting pharmacological target to regulate cardiovascular cell apoptosis, proliferation, inflammation, and metabolism.

## 1. Introduction

Peroxisome proliferator activated receptors (PPARs) are transcriptional factors of intense interest through their involvement in several biological processes such as energy homeostasis, cell proliferation and differentiation, fatty acid catabolism, and adipogenesis [1]. Among the three PPAR isotypes identified, PPAR*α* and PPAR*γ* had been the most extensively studied. PPAR*α* is activated by several fatty acids and expressed in tissues exhibiting a high rate of fatty acid catabolism (liver, heart, kidney, and muscle). In the cardiovascular system, PPAR*α* is expressed in cardiac and smooth muscle cells, in endothelial cells and monocyte/macrophage cells [2, 3], and it regulates fatty acid transport, esterification, and oxidation, via activation of genes encoding key enzymes involved in these processes [4]. We have also previously described that the PPAR*α* gene deletion induced defects of the cardiac contractile performance and myocardial fibrosis, suggesting a major role of PPAR*α* in the maintenance of cardiovascular homeostasis within the physiological range [5].

PPAR*γ* is the most studied PPAR subtype, and is primarily expressed in adipose tissue where it participates in the transcription of various genes involved in lipid metabolism, glucose homeostasis regulation, and in the inflammatory processes, preventing foam cell formation [6] and reducing nitric oxide (NO) overproduction, interleukin-6 (IL-6), and tumor necrosis factor-*α* (TNF-*α*) expressions. PPAR*γ* activation suppresses cyclo-oxygenase-2 (COX-2)- and inducible nitric oxide synthase (iNOS)-inductions by repression of NF-*κ*B and AP-1 [7, 8]. PPAR*γ* can be activated by synthetic agonists of the thiazolidinedione family, for instance, rosiglitazone (RZ) [9], which is able to reduce the inflammatory markers in diabetic patients [10]. Several studies have shown that some effects of PPAR*γ* agonists are PPAR*γ*-independent [11, 12]. In contrast, we have recently found a direct PPAR*γ*-dependent protective action of RZ on vascular dysfunction accompanying smooth muscle cell inflammation. Indeed, selective PPAR*γ* inhibitor, GW9662, completely abolished the beneficial anti-inflammatory effects of RZ [13].

PPAR*δ* (also known as PPAR*β* and NR1C2) is the most ubiquitously expressed, although its physiological and pathological roles are unclear, especially in human tissues [14, 15]. Prostacyclin, the predominant prostanoid released by vascular cells, is a putative endogenous agonist for PPAR*δ* [16, 17]. The roles of PPAR*α* and PPAR*γ* in vascular cells were extensively investigated in a variety of in vitro and in vivo models, both having in general anti-inflammatory and antiproliferative properties [18]. In contrast, the role of PPAR*δ* in cardiovascular functions is not fully understood but there is a rising interest for this nuclear receptor in this domain because of its pivotal role in apoptosis and cell proliferation [19–21], and for its function as a key regulator of fatty acid metabolism [22, 23]. On the light of the recent data, in the present review, we focused our attention on the new therapeutic approaches, using selective agonists of PPAR*δ*, to regulate cardiovascular function and cardiac and vascular cell proliferations and apoptosis in several cardiovascular pathologies.

## 2. PPAR*δ* Agonists

Like other PPAR subtypes, PPAR*δ* is activated by a large variety of endogenous agonists, such as lipids, including long-chain dietary fatty acids [23], and prostacyclin (PGI_2_) [16, 17]. Synthetic analogs of PGI_2_, such as carbaprostacyclin (cPGI_2_), are able to bind to and to act as agonists of PPAR*δ*. PPAR*α* and PPAR*δ* possess overlapping ligand specificities, thus, fatty acids and PGI_2_ analogs have been shown to induce transcriptional activation and DNA binding of both PPAR subtypes [24]. Moreover, stable prostacyclin analogs are able to activate in vitro also PPAR*γ* in a cell surface prostacyclin receptor-dependent manner [25].

Moreover, several high-affinity synthetic PPAR*δ* ligands were identified and have contributed to improve studies about the physiological and pathophysiological roles of PPAR*δ* activation. These PPAR*δ* activators include the phenoxyacetic acid derivatives GW501516 and GW0742 (GlaxoSmithKline, Brentford, UK) [26], and L165041 (Merck, Whitehouse Station, NJ, USA) [27]. GW501516 and GW0742 agonists show a thousand-fold PPAR*δ* selectivity over other PPAR subtypes [26] while L165041 has a weak PPAR*γ* binding activity at concentrations higher than 5 *μ*M [27]. The PPAR*δ* agonists have different bioavailability and effects in the lipoprotein delivery system according to animal models, primate, or mouse for instance [28]. There are also two other synthetic agonists with high affinity for both PPAR*γ* and PPAR*δ*: L796449 and L165461 [27]. Now, several new synthetic PPAR*δ* agonists are in development for clinical applications: MBX-8025 (Metabolex Inc, Calif, USA), CER-002 (Cerenis Therapeutics, Mich, USA), and KD3010 (Kalypsys, Calif, USA) [29].

## 3. Role of PPAR*δ* in Cell Apoptosis and Cell Proliferation

Several reports showed that PPAR*δ* activation by PGI_2_, its analogues, or selective ligands, acutely regulates endothelial cell apoptosis and protects endothelial cells from apoptosis caused by oxidant stress [30], or induces human umbilical endothelial cell (HUVEC) proliferation [19]. PPAR*δ* activity is also involved in a negative control of colorectal cancer and keratinocyte cell apoptosis [31, 32]. Synthetic PGI_2_ has been shown to protect renal cells from hypertonicity-induced apoptosis, which was attributed to PPAR*δ* activation [33]. More recently, it was found that endothelial cell survival was improved in HUVECs transduced with an adenovirus containing genes that selectively increased PGI_2_ synthesis [30]. The authors revealed, for the first time, that PGI_2_ upregulates 14-3-3*ε* promoter activity in a PPAR*δ*-dependent manner. Indeed, 14-3-3*ε* upregulation leads to increase of BAD binding and decreasing of BAD translocation to mitochondria with subsequent inhibition of cytochrome c release, caspase-3 activation and endothelial cell hydrogen peroxide (H_2_O_2_)-induced apoptosis [30]. Furthermore, the PPAR*δ* overexpression amplifies the antiapoptotic action of PGI_2_. More recently, the mechanisms by which PPAR*δ* agonists control the 14-3-3*ε* expression were investigated and it was suggested a key anti-inflammatory role of PPAR*δ* activation in promoting endothelial cell survival [34].

It is known that reactive oxygen species (ROS), mediators of oxidative stress, play a deleterious role on vasculature and myocardium. ROS and H_2_O_2_ have been reported to induce apoptosis in various cell types. The role of PPAR*δ* activation using GW501516 was also investigated in H_2_O_2_-induced apoptosis in rat cardiomyoblasts. PPAR*δ* exerts an antioxidant role in rat cardiac myoblasts by increasing catalase expression in presence of H_2_O_2_ with a subsequent cell protection from H_2_O_2_-induced apoptosis that is caspase-3-dependent [35].

Concerning the cardiac injury and cardiomyocyte death induced by ischemia, a recent study provides several lines of evidence indicating a cardioprotective effect of GW0742, via upregulation of survival signalling and suppression of apoptotic cell death. Indeed, GW0742 is able to reverse the detrimental effect of ischemia/reperfusion on expression of Bcl family genes. This reduces expression of proapoptotic genes, Bax and Bid, and reverses the ischemia-dependent downregulation of the antiapoptotic gene, Bcl-xL [36]. Moreover, GW0742 treatment reduces the ischemia/reperfusion-induced cardiac mitochondrial damage that is a critical step in cell apoptotic death controlled by Bcl family proteins. Activation of PPAR*δ* by this agonist also reduces the myocardial caspase-3 activity and increases cardiac p-Akt and its upstream regulator, p-PDK, a central kinase for signalling pathway regulating cell survival and cell growth [37].

**Figure 1:**
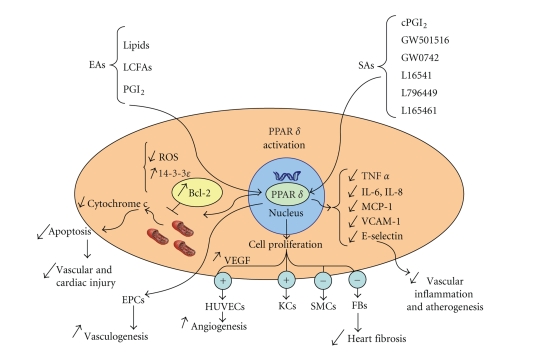
Effects of PPAR*δ* activation on potential targets implicated in cell death, cell proliferation, and inflammation. PPAR*δ* can be activated not selectively by endogenous agonists (EAs), such as several lipids, long-chain fatty acids (LCFAs), and prostacyclin (PGI_2_), or synthetic agonists (SAs). PPAR*δ* activation has an antiapoptotic effect reducing reactive oxygen species (ROS), upregulating 14-3-3*ε* activity and reducing cytochrome c release from mitochondria and subsequent caspase-3 activation. The effect of PPAR*δ* activation on cell proliferation is cell type dependent. It has a proangiogenetic effect in human umbilical endothelial cells (HUVECs), a proproliferative effect in keratinocytes (KCs), while an antiproliferative effect in smooth muscle cells (SMCs) and in cardiac fibroblasts (FBs) with subsequent reduction of heart fibrosis. PPAR*δ* activation in endothelial progenitor cells (EPCs) enhances vasculogenesis. PPAR*δ* has anti-inflammatory effects by reducing cytokines production and molecules implicated in endothelial/leukocyte interactions.

The role of PPAR*δ* activation in cell proliferation is somewhat controversial. Cardiac fibroblast proliferation, differentiation of fibroblasts to myofibroblasts, and collagen synthesis were reduced after PPAR*δ* activation [37]. It was shown that a prostacyclin agonist, the beraprost sodium, is able to enhance the PPAR*δ* and iNOS expressions with an antiproliferative effect in rat aortic smooth muscle cells, suggesting that iNOS is a downstream target of PPAR*δ* [38]. Moreover, suppression of PPAR*δ* expression by RNAi targeting significantly promoted the proliferation of human colorectal cancer cells (HCT-116) by increasing the number of cells in G1 phase [39]. In contrast, in another work, Zeng et al. have shown that PPAR*δ* is able to induce cell proliferation in human thyroid tumors by regulating epithelial cell proliferation via cyclin E1, growth factor and lipid signals. Moreover, PPAR*δ* is upregulated in human thyroid tumors and the deregulation of the PPAR*δ*/cyclin E1 pathway may be important not only in thyroid cancer but also in other types of carcinomas [40]. PPAR*δ* expression and activation were rapidly stimulated by epidermal growth factor (EGF) stimuli in HaCaT keratinocytes and this promoted cell proliferation [41]. PPAR*δ*-/- mice exhibit increased keratinocyte proliferation when treated with a tumor promoter [42]. On the light of these new studies, the effects of PPAR*δ* activation on cell proliferation seem to be cell type specific. PPAR*δ* roles in apoptosis and cell proliferation are synthesized in Figure 1.

## 4. PPAR*δ* in Angiogenesis and Vasculogenesis

PPAR*δ* activation could play an important role also in vascular growth, in particular in vasculogenesis from endothelial progenitor cells (EPCs) and angiogenesis deriving from pre-existing endothelial cells [43]. A recent study showed that PPAR*δ* activation, using GW501516, induces cell proliferation in human endothelial cell cultures and angiogenesis in a murine Matrigel plug assay in vivo through a mechanism that involves vascular endothelium growth factor (VEGF) gene transcription. Moreover, the same study showed that GW501516 is able to induce transcription of PPAR*δ* target genes such as the adipose differentiation-related protein (ADRP), VEGF, and the matrix metalloproteinase-9. These data suggest that endogenous prostacyclin, like the synthetic agonist GW501516, may regulate endothelial cell proliferation and angiogenesis through a PPAR*δ*-dependent VEGF gene expression [19].

PPAR*δ* activation with agonists, GW501516 or L-165041, increased the proliferation of human early EPCs and protected them from hypoxia-induced apoptosis. In addition, PPAR*δ* activation enhanced EPC transendothelial migration and tube formation [44]. Injection of the GW501516-treated human or mouse early EPCs, in a hind limb ischemia model of athymic nude mice, significantly accelerated limb perfusion improvement after 21 days of ischemia. This was associated with an increased capillary density determined by histological studies. PPAR*δ* agonist-treated EPCs from PPAR-*δ*-/- mice failed to enhance vasculogenesis. These findings suggest that ex vivo activation of PPAR*δ* in early EPCs results in enhanced vasculogenic potential in vivo, and this was confirmed also in a corneal neovascularization model [43]. The beneficial effects observed after PPAR*δ* activation in EPCs are mediated by the PIK/Akt pathway implicated in vasculogenesis [45–47]. More recently, He and coworkers provided evidences that the proangiogenic effects of human late EPCs are partially dependent on biosynthesis and release of PGI_2_ and subsequent PPAR*δ* activation [48]. Indeed, in the same study, the downregulation of PPAR*δ* expression impaired in vivo angiogenesis in nude mice transplanted with EPCs, treated with COX-1 or PGI_2_ synthase (PGIS) small interfering RNAs, supporting the concept that the COX-1/PGI_2_/PPAR*δ* pathway in human EPCs plays an important role in angiogenesis [48].

Interestingly, a tumor vascularization defect was found in PPAR*δ*-/- mice with subsequent decreased progression and partial regression of tumors in this mouse model. This was due to the abundance of highly abnormal hyperplastic microvessels appearing dysfunctional in tumors from PPAR*δ*-/- mice [49]. Even though a defect in angiogenesis has not been observed during normal development of PPAR*δ*-/- mice, PPAR*δ* is specifically required by tumor endothelial cells to orchestrate their proliferation and differentiation in an environment providing abnormal sources of growth factors and cytokines [49]. These findings suggest an interesting potential for clinical applications of PPAR*δ* as a target in the tumor treatment.

## 5. Role of PPAR*δ* in Atherosclerosis

The atheroprotective role of PPAR*δ* activation remains controversial. The PPAR*δ* activation using GW0742 reduces only weakly atherosclerosis formation in mice knockout for the low-density lipoprotein receptor (LDLR) gene [50]. In another study, administration of the same agonist at a lower dose failed to have an effect in foam cell formation and in vascular lesions in the same mouse model [51]. The limited atheroprotective effect of GW0742 might be explained through its inability to modify high-density lipoprotein cholesterol (HDL-c) levels in both studies.

Besides, more recently, it was confirmed a vascular protective effect of PPAR*δ* activation in a model of angiotensin II (Ang II)-induced atherosclerosis in LDLR-/- mice. The authors evidenced attenuated Ang II-accelerated atherosclerosis through the increased expression of the anti-inflammatory corepressor, B cell lymphoma-6 (Bcl-6). Furthermore, Ang II activation of MAP kinases (p38 and ERK1/2) was inhibited in LDLR-/- mice infused with Ang II and treated with a high-fat diet supplemented with GW0742 [52]. Another selective PPAR*δ* agonist, GW501516, has been reported to reduce atherosclerotic lesion formation in ApoE-/- mice submitted to high-fat diet [53]. This agonist was able to improve HDL-c blood levels in these mice in agreement with results previously obtained in monkeys [54]. Indeed, GW501516 promotes reverse cholesterol transport in macrophages, a key step in reducing foam cell formation, by increasing expression of the reverse cholesterol ATP-binding cassette transporter A1. This agonist also causes a dramatic dose-dependent rise of HDL-c, while it decreases the levels of LDLs in serum of obese Rhesus monkeys [54]. PPAR*δ* agonists may also possess antiatherosclerotic properties in vivo by decreasing the amount of nonliganded PPAR*δ* receptor and by releasing the transcriptional repressor Bcl-6 of foam cells [55]. Furthermore, in the work of Barish et al. [53], DNA array and real-time PCR analyses, conducted in aortas from GW501516-treated ApoE-/- mice, have evidenced potential PPAR*δ* target genes. Experiences have shown a downregulation of both CXCL7 and CCL21*α*, implicated in neutrophil and T lymphocyte recruitment induction. In contrast, others proteins were upregulated, in particular the regulators of G protein signalling (RGS) 4 and 5, which antagonize the signalling mediated by the chemokine receptors. The tissue inhibitor of metalloproteinase 3 (TIMP3), implicated in smooth muscle cell migration and lesion stability, was also upregulated. At last, chemokine expression was downregulated by GW501516, confirming the anti-inflammatory property of PPAR*δ* activation [53].

Endothelial cell monolayer is exposed to circulating inflammatory factors that predispose to atherosclerosis. Increasing evidences suggest that the endothelial cell apoptosis might contribute to the development of atherosclerosis and acute coronary syndromes [55]. PPAR*δ* agonists might modulate the development of atherosclerosis and acute coronary syndrome, not only by targeting foam cells and lipoprotein metabolism [56, 57], or protecting against obesity [58], but also by promoting endothelial cell survival via 14-3-3*ε* [34]. Indeed, 14-3-3 and 14-3-3*ε* proteins are antiapoptotic and anti-inflammatory molecules in endothelial cells and they may play an important role in atherothrombosis [30, 59, 60]. In particular, 14-3-3 proteins modulate crucial aspects of heart function both in and in vivo [60–63]. In a recent study, GW0742 and GW501516 significantly inhibit the vascular cell adhesion molecule 1 (VCAM-1) and E-selectin expressions induced by TNF*α*, ensuing in reduced endothelial-leukocyte adhesion in HUVECs. Moreover, PPAR*δ* activation upregulates expression of antioxidative genes such as superoxide dismutase 1, catalase, and thioredoxin, leading to a reduced ROS production in endothelial cells [64]. These studies suggest the protective role played by PPAR*δ* in the regulation of multiple proinflammatory pathways with subsequent atherosclerosis suppression.

## 6. Role of PPAR*δ* in Cardiac Protection

PPAR*δ* is the predominant PPAR subtype in cardiac cells and it is implicated in the regulation of cardiac lipid metabolism, suggesting its important role in cardiac diseases. Indeed, mice with cardiac-specific deletion of the PPAR*δ* gene developed myocardial lipid accumulation and cardiomyopathy [65].

The inflammatory response following cardiac ischemia/reperfusion is a determinant factor responsible for tissue injury development triggered by the cytokine cascade and the upregulation of adhesion molecules and chemokines. These events enhanced neutrophil and monocyte infiltrations into myocardium and lead to cardiac damage [66]. The PPAR*δ* activation by GW0742 reduces inflammatory cytokine expression, ending up in a reduction of plasma levels of some interleukins (IL-6 and IL-8). Furthermore, GW0742 is able to decrease expression of the adhesion molecule for leukocytes, ICAM-1, and of the chemokine responsible for monocyte recruitment, MCP-1, in a cardiac ischemia/reperfusion model [36].

Diabetes predisposes to heart failure, particularly in combination with other comorbid conditions such as hypertension and coronary artery disease [67]. The incidence of heart failure and death, following myocardial infarction, is higher in diabetic than in nondiabetic individuals [68]. PPAR*α* and PPAR*δ* drive distinct cardiac metabolic regulatory programs in mouse models of type 2 diabetes. Indeed, PPAR*δ* activates, whereas PPAR*α* represses, targets involved in the cellular glucose utilization, resulting in reciprocal effects on cellular glucose uptake through differential regulation of glucose transporter 4 (GLU4/SLC2A4) transcription. These findings suggest that cardiac specific overexpression of PPAR*δ* is cardioprotective in diabetes [22].

The development of heart failure is also associated with extensive fibrosis, which aggravates diastolic dysfunction and predisposes to arrhythmias [69, 70]. A critical event in fibrosis initiation is the proliferation and differentiation of cardiac fibroblasts. Upon activation by cytokines, growth factors, or stretch, fibroblasts start their proliferation and ultimately differentiate towards myofibroblasts, acquiring smooth muscle-like properties [71, 72]. Although fibroblasts take care of normal collagen turnover [73], myofibroblasts are rather responsible for the alteration of extracellular matrix accumulation, often leading to impaired organ function [54]. PPAR*δ* activation leads to reduced cardiac fibroblast proliferation by a mechanism that involves the upregulation of PPAR-responsive cell cycle inhibitory G0S2 gene [37]. Moreover, heart collagen is inhibited after PPAR*δ* activation. These findings are in agreement with another work, in which it was found a protective effect of PPAR*δ* activation by GW501516 in the inhibition of Ang II-induced collagen type I expression via a decreased collagen synthesis in adult rat cardiac fibroblasts [74].

In the transitions from foetal to neonatal and to adult life, cardiac metabolism switches from glucose to fatty acids as a preferred energetic substrate to generate ATP [75]. In contrast, cardiac hypertrophy is associated with an increase in glucose utilization and a decrease in fatty acid oxidation [76]. It was found that the PPAR*δ* activation by L-165041 inhibits the phenylephrine-induced hypertrophy in neonatal rat cardiac myocytes. The target is NF-*κ*B signalling pathway that plays a pivotal role in the hypertrophic response of cultured cardiac myocytes [77]. Moreover, hypertrophy in neonatal rat cardiomyocytes caused a reduction in expression of pyruvate dehydrogenase kinase 4 (*Pdk4*), a target gene of PPAR*δ* involved in fatty acid utilization. Indeed, NF-*κ*B activation, during cardiac hypertrophy, downregulates PPAR*δ* activity, leading to a fall in fatty acid oxidation, through a mechanism that involves enhanced protein-protein interactions between the p65 subunit of NF-*κ*B and PPAR*δ* and a subsequent reduction on expression of PPAR*δ* target genes [23]. On the basis of the literature, one can think that several therapeutic approaches using PPAR*δ* agonists may be possible to treat vascular and cardiac pathological states, especially those in which inflammation, fibrosis, and lipid metabolic disorders are involved. The effects of PPAR*δ* activation on cardiac cells are synthesized in Figure 1.

## 7. Role of PPAR*δ* Activation in Metabolic Diseases

High-fat diet, a sedentary way of life, and genetic factors seem to account for the development of cardiovascular diseases, including atherosclerosis and heart stroke. At the same time, obesity is an important risk factor for cardiovascular disorders, since it is often associated with hypertension and increases the risk of metabolic disorders such as insulin resistance, hypertriglyceridemia and low plasmatic levels of HDL-c. Patients characterized by these metabolic abnormalities may be considered as affected by the metabolic syndrome. PPAR*δ* is involved in lipid metabolism control and energy homeostasis [78]. The PPAR*δ* activation promotes fatty acid catabolism in several tissues, such as skeletal muscles and adipose tissue [57, 79]. Moreover, several recent studies suggest a potential role of PPAR*δ* in the regulation of glucose metabolism and insulin sensitivity [80]. PPAR*δ* agonists have advantageous effects in obesity prevention and modulation of lipoprotein metabolism [54, 57, 58]. Indeed, transgenic mice overexpressing PPAR*δ* are protected against diet-induced obesity through an increased catabolism of fatty acids [58]. Moreover, administration of PPAR*δ* agonists to mice treated with a high-fat diet decreased insulin resistance by enhancing fatty acid oxidation and decreasing lipid content of skeletal muscles [57].

Pharmacological activation of PPAR*δ*, using GW0742, protects heart from ischemia/reperfusion injury in male Zucker fatty rats, a rodent model of obesity and dyslipidemia [36]. All these studies suggest a protective effect of PPAR*δ* activation in the cardiovascular complications induced by metabolic disorders.

## 8. Conclusion

PPAR*δ* activation is presently used as a new therapeutic approach in several metabolic and cardiovascular pathological states. PPAR*δ* activators are in development for the treatment of dyslipidemia, obesity and/or insulin resistance in patients with the metabolic syndrome [78]. PPAR*δ* agonists have advantageous effects in obesity prevention and modulation of lipoprotein metabolism [54, 57, 58]. GW501516 was tested for its effects on dyslipidemia in obese Rhesus monkeys. This agonist increased of 79% HDL-c and decreased of 56% and of 29% plasmatic levels of triglycerides and LDL-c, respectively [54]. The same agonist increased HDL-c, apolipoprotein A-1, and apolipoprotein A-2 in vervet monkeys, a primate atherosclerosis model [81]. The results of the first human trial, conducted with a small cohort of healthy volunteers, have shown no toxicity during a treatment period of two weeks with GW501516 at the doses used for this study [82]. GW501516 has been in a phase II clinical safety/efficacy study for dyslipidemia, completed in October 2008 by GlaxoSmithKline. The results of this study have not been published yet. The use of GW501516 could be also a promising therapeutic approach to prevent multiple aspects of the cardiac fibrotic process [37, 60]. PPAR*δ* activation and/or overexpression, improving increased glucose use in diabetic heart, shows promises as a therapeutic strategy for cardiac dysfunction caused by diabetes and ischemia [22]. Furthermore, the use of GW0742 may be a new pharmacological approach to protect patients affected by metabolic syndrome from their elevated risk of ischemic heart disease. GW0742 decreases lipotoxicity, inflammation, and upregulates cell survival [36]. GW0742 and GW501516 could be also used to treat inflammatory diseases such as atherosclerosis and diabetes [64].

MBX-8025 is one of the most advanced PPAR*δ* agonists currently in a phase II clinical trial for dyslipidemia and in particular for its effects on lipid and metabolic parameters, including triglycerides, LDL and HDL-c, insulin sensitivity, and inflammation. This drug was administrated alone or in association with Lipitor (atorvastatin, Pfizer Inc., NY, USA), to patients affected by metabolic syndrome. MBX-8025 depleted substantially the small, dense, LDL-cholesterol particles, alone or in combination with Lipitor. Other second-generation PPAR*δ* agonists, CER-002 and KD3010, are in phase I clinical trial for atherosclerosis and metabolic disorder treatments, respectively [29].

Recombinant adenovirus, bearing a gene that selectively increases PGI_2_ synthesis and subsequent PPAR*δ* activation, has been shown to protect neurons from ischemia/reperfusion in vivo in a model of ischemic cerebral infarction [83]. This could be attributed to the antiapoptotic action of PGI_2_. Gene transfer may be used as a new potential treatment for protecting blood vessels during ischemic diseases through reduction of the vascular cell death.

On the light of these recent findings, we can affirm that PPAR*δ* regulation by appropriate selective agonists, as well as PPAR*α* and PPAR*γ* activation, could be used as a novel therapeutic intervention in metabolic and cardiovascular inflammatory diseases through its effect in atherogenesis control and angiogenesis regulation. Nevertheless, the long-term use of PPAR*δ* ligands in patients susceptible to angiogenic diseases, such as diabetics who are prone to retinopathy or individuals predisposed to cancer, may require particular care and a better understanding of its possible collateral effects.

## References

[1] Escher P, Wahli W (2000). Peroxisome proliferator-activated receptors: insight into multiple cellular functions. *Mutation Research/Fundamental and Molecular Mechanisms of Mutagenesis*.

[2] Issemann I, Green S (1990). Activation of a member of the steroid hormone receptor superfamily by peroxisome proliferators. *Nature*.

[3] Kersten S, Desvergne B, Wahli W (2000). Roles of PPARs in health and disease. *Nature*.

[4] Barger PM, Kelly DP (2000). PPAR signaling in the control of cardiac energy metabolism. *Trends in Cardiovascular Medicine*.

[5] Loichot C, Jesel L, Tesse A (2006). Deletion of peroxisome proliferator-activated receptor-*α* induces an alteration of cardiac functions. *American Journal of Physiology*.

[6] Son N-H, Park T-S, Yamashita H (2007). Cardiomyocyte expression of PPAR*γ* leads to cardiac dysfunction in mice. *The Journal of Clinical Investigation*.

[7] Inoue H, Tanabe T, Umesono K (2000). Feedback control of cyclooxygenase-2 expression through PPAR*γ*. *The Journal of Biological Chemistry*.

[8] Maggi LB, Sadeghi H, Weigand C, Scarim AL, Heitmeier MR, Corbett JA (2000). Anti-inflammatory actions of 15-deoxy-Δ^12,14^-prostaglandin J_2_ and troglitazone evidence for heat shock-dependent and -independent inhibition of cytokine-induced inducible nitric oxide synthase expression. *Diabetes*.

[9] Mohanty P, Aljada A, Ghanim H (2004). Evidence for a potent antiinflammatory effect of rosiglitazone. *The Journal of Clinical Endocrinology & Metabolism*.

[10] Marfella R, D'Amico M, Esposito K (2006). The ubiquitin-proteasome system and inflammatory activity in diabetic atherosclerotic plaques: effects of rosiglitazone treatment. *Diabetes*.

[11] Hinz B, Brune K, Pahl A (2003). 15-deoxy-Δ^12,14^-prostaglandin J_2_ inhibits the expression of proinflammatory genes 
in human blood monocytes via a PPAR-*γ*-independent mechanism. *Biochemical and Biophysical Research Communications*.

[12] Okura T, Nakamura M, Takata Y, Watanabe S, Kitami Y, Hiwada K (2000). Troglitazone induces apoptosis via the p53 and Gadd45 pathway in vascular smooth muscle cells. *European Journal of Pharmacology*.

[13] Tesse A, Al-Massarani G, Wangensteen R, Reitenbach S, Martínez MC, Andriantsitohaina R (2008). Rosiglitazone, a peroxisome proliferator-activated receptor-*γ* agonist, prevents microparticle-induced vascular hyporeactivity through the regulation of proinflammatory proteins. *The Journal of Pharmacology and Experimental Therapeutics*.

[14] Kliewer SA, Forman BM, Blumberg B (1994). Differential expression and activation of a family of murine peroxisome proliferator-activated receptors. *Proceedings of the National Academy of Sciences of the United States of America*.

[15] Mukherjee R, Jow L, Croston GE, Paterniti JR (1997). Identification, characterization, and tissue distribution of human peroxisome proliferator-activated receptor (PPAR) isoforms PPAR*γ*2 *versus* 
PPAR*γ*1 and activation with retinoid X receptor agonists and antagonists. *The Journal of Biological Chemistry*.

[16] Gupta RA, Tan J, Krause WF (2000). Prostacyclin-mediated activation of peroxisome proliferator-activated receptor *δ* in colorectal cancer. *Proceedings of the National Academy of Sciences of the United States of America*.

[17] Pola R, Gaetani E, Flex A (2004). Comparative analysis of the in vivo angiogenic properties of stable 
prostacyclin analogs: a possible role for peroxisome proliferator-activated receptors. *Journal of Molecular and Cellular Cardiology*.

[18] Moraes LA, Piqueras L, Bishop-Bailey D (2006). Peroxisome proliferator-activated receptors and inflammation. *Pharmacology and Therapeutics*.

[19] Piqueras L, Reynolds AR, Hodivala-Dilke KM (2007). Activation of PPAR*β*/*δ* induces endothelial cell proliferation and angiogenesis. *Arteriosclerosis, Thrombosis, and Vascular Biology*.

[20] Fukumoto K, Yano Y, Virgona N (2005). Peroxisome proliferator-activated receptor *δ* as a molecular target to regulate lung cancer cell growth. *FEBS Letters*.

[21] Stephen RL, Gustafsson MCU, Jarvis M (2004). Activation of peroxisome proliferator-activated receptor *δ* stimulates the proliferation of human breast and prostate cancer cell lines. *Cancer Research*.

[22] Burkart EM, Sambandam N, Han X (2007). Nuclear receptors PPAR*β*/*δ* and PPAR*α* direct distinct metabolic regulatory programs in the mouse heart. *The Journal of Clinical Investigation*.

[23] Planavila A, Laguna JC, Vázquez-Carrera M (2005). Nuclear factor-*κ*B activation leads to down-regulation of fatty acid oxidation during cardiac hypertrophy. *The Journal of Biological Chemistry*.

[24] Forman BM, Chen J, Evans RM (1997). Hypolipidemic drugs, polyunsaturated fatty acids, and eicosanoids are 
ligands for peroxisome proliferator-activated receptors *α* and *δ*. *Proceedings of the National Academy of Sciences of the United States of America*.

[25] Falcetti E, Flavell DM, Staels B, Tinker A, Haworth SG, Clapp LH (2007). IP receptor-dependent activation of PPAR*γ* 
by stable prostacyclin analogues. *Biochemical and Biophysical Research Communications*.

[26] Sznaidman ML, Haffner CD, Maloney PR (2003). Novel selective small molecule agonists for peroxisome proliferator-activated 
receptor *δ* (PPAR*δ*)-synthesis and 
biological activity. *Bioorganic & Medicinal Chemistry Letters*.

[27] Berger J, Leibowitz MD, Doebber TW (1999). Novel peroxisome proliferator-activated receptor (PPAR) *γ* and PPAR*δ* ligands produce distinct biological effects. *The Journal of Biological Chemistry*.

[28] Kang K, Hatano B, Lee C-H (2007). PPAR*δ* agonists and metabolic diseases. *Current Atherosclerosis Reports*.

[29] Rittenhouse PA (2008). Dyslipidemia rescue. *BioCentury, The Bernstein Report on BioBusiness*.

[30] Liou J-Y, Lee S, Ghelani D, Matijevic-Aleksic N, Wu KK (2006). Protection of endothelial survival by peroxisome proliferator-activated receptor-*δ* mediated 14-3-3 upregulation. *Arteriosclerosis, Thrombosis, and Vascular Biology*.

[31] He T-C, Chan TA, Vogelstein B, Kinzler KW (1999). PPAR*δ* is an APC-regulated target of nonsteroidal anti-inflammatory drugs. *Cell*.

[32] Tan NS, Michalik L, Noy N (2001). Critical roles of PPAR*β*/*δ* in keratinocyte response to inflammation. *Genes & Development*.

[33] Hao C-M, Redha R, Morrow J, Breyer MD (2002). Peroxisome proliferator-activated receptor *δ* activation promotes cell survival following hypertonic stress. *The Journal of Biological Chemistry*.

[34] Brunelli L, Cieslik KA, Alcorn JL, Vatta M, Baldini A (2007). Peroxisome proliferator-activated receptor-*δ* 
upregulates 14-3-3*ε* in human endothelial cells via CCAAT/enhancer binding protein-*β*. *Circulation Research*.

[35] Pesant M, Sueur S, Dutartre P (2006). Peroxisome proliferator-activated receptor *δ* 
(PPAR*δ*) activation protects H9c2 cardiomyoblasts from oxidative stress-induced apoptosis. *Cardiovascular Research*.

[36] Yue T-L, Nerurkar SS, Bao W (2008). In vivo activation of peroxisome proliferator-activated receptor-*δ* protects the heart from ischemia/reperfusion injury in Zucker fatty rats. *The Journal of Pharmacology and Experimental Therapeutics*.

[37] Teunissen BEJ, Smeets PJH, Willemsen PHM, De Windt LJ, Van der Vusse GJ, Van Bilsen M (2007). Activation of PPAR*δ* inhibits cardiac fibroblast proliferation and the transdifferentiation into myofibroblasts. *Cardiovascular Research*.

[38] Lin H, Lee J-L, Hou H-H, Chung C-P, Hsu S-P, Juan S-H (2008). Molecular mechanisms of the antiproliferative effect of beraprost, a prostacyclin agonist, in murine vascular smooth muscle cells. *Journal of Cellular Physiology*.

[39] Yang L, Zhou Z-G, Zheng X-L (2008). RNA interference against peroxisome proliferator-activated receptor *δ* gene promotes proliferation of human colorectal cancer cells. *Diseases of the Colon and Rectum*.

[40] Zeng L, Geng Y, Tretiakova M, Yu X, Sicinski P, Kroll TG (2008). Peroxisome proliferator-activated receptor-*δ* induces cell proliferation by a cyclin E1-dependent mechanism and is up-regulated in thyroid tumors. *Cancer Research*.

[41] Liang P, Jiang B, Yang X (2008). The role of peroxisome proliferator-activated receptor-*β*/*δ* in epidermal growth factor-induced HaCaT cell proliferation. *Experimental Cell Research*.

[42] Michalik L, Desvergne B, Tan NS (2001). Impaired skin wound healing in peroxisome proliferator-activated receptor (PPAR)*α* and PPAR*β* mutant mice. *Journal of Cell Biology*.

[43] Semenza GL (2007). Vasculogenesis, angiogenesis, and arteriogenesis: mechanisms of blood vessel formation and remodeling. *Journal of Cellular Biochemistry*.

[44] Han JK, Lee HS, Yang HM (2008). Peroxisome proliferator-activated receptor-*δ* agonist enhances vasculogenesis by regulating endothelial progenitor cells through genomic and nongenomic activations of the phosphatidylinositol 3-kinase/Akt pathway. *Circulation*.

[45] Hur J, Yoon C-H, Lee C-S (2007). Akt is a key modulator of endothelial progenitor cell trafficking in ischemic muscle. *Stem Cells*.

[46] Dimmeler S, Aicher A, Vasa M (2001). HMG-CoA reductase inhibitors (statins) increase endothelial progenitor cells via the PI 3-kinase/Akt pathway. *The Journal of Clinical Investigation*.

[47] Llevadot J, Murasawa S, Kureishi Y (2001). HMG-CoA reductase inhibitor mobilizes bone marrow-derived endothelial progenitor cells. *The Journal of Clinical Investigation*.

[48] He T, Lu T, d'Uscio LV, Lam CF, Lee HC, Katusic ZS (2008). Angiogenic function of prostacyclin biosynthesis in human endothelial progenitor cells. *Circulation Research*.

[49] Müller-Brüsselbach S, Kömhoff M, Rieck M (2007). Deregulation of tumor angiogenesis and blockade of tumor growth in PPAR*β*-deficient mice. *The EMBO Journal*.

[50] Graham TL, Mookherjee C, Suckling KE, Palmer CNA, Patel L (2005). The PPAR*δ* agonist GW0742X 
reduces atherosclerosis in LDLR^−/−^ mice. *Atherosclerosis*.

[51] Li AC, Binder CJ, Gutierrez A (2004). Differential inhibition of macrophage foam-cell formation and atherosclerosis in mice by PPAR*α*, *β*/*δ*, and *γ*. *The Journal of Clinical Investigation*.

[52] Takata Y, Liu J, Yin F (2008). PPAR*δ*-mediated antiinflammatory mechanisms inhibit angiotensin II-accelerated atherosclerosis. *Proceedings of the National Academy of Sciences of the United States of America*.

[53] Barish GD, Atkins AR, Downes M (2008). PPAR*δ* regulates multiple proinflammatory 
pathways to suppress atherosclerosis. *Proceedings of the National Academy of Sciences of the United States of America*.

[54] Oliver WR, Shenk JL, Snaith MR (2001). A selective peroxisome proliferator-activated receptor *δ* agonist promotes reverse cholesterol transport. *Proceedings of the National Academy of Sciences of the United States of America*.

[55] Dimmeler S, Haendeler J, Zeiher AM (2002). Regulation of endothelial cell apoptosis in atherothrombosis. *Current Opinion in Lipidology*.

[56] Lee C-H, Chawla A, Urbiztondo N, Liao D, Boisvert WA, Evans RM (2003). Transcriptional repression of atherogenic inflammation: modulation by PPAR*δ*. *Science*.

[57] Tanaka T, Yamamoto J, Iwasaki S (2003). Activation of peroxisome proliferator-activated receptor *δ* induces fatty acid *β*-oxidation in skeletal muscle and attenuates metabolic syndrome. *Proceedings of the National Academy of Sciences of the United States of America*.

[58] Wang Y-X, Lee C-H, Tiep S (2003). Peroxisome-proliferator-activated receptor *δ* activates fat metabolism to prevent obesity. *Cell*.

[59] Liu Y, Yin G, Surapisitchat J, Berk BC, Min W (2001). Laminar flow inhibits TNF-induced ASK1 activation by preventing dissociation of ASK1 from its inhibitor 14-3-3. *The Journal of Clinical Investigation*.

[60] Zhang S, Ren J, Zhang CE, Treskov I, Wang Y, Muslin AJ (2003). Role of 14-3-3-mediated p38 mitogen-activated protein kinase inhibition in cardiac myocyte survival. *Circulation Research*.

[61] Kagan A, Melman YF, Krumerman A, McDonald TV (2002). 14-3-3 amplifies and prolongs adrenergic stimulation of HERG K^+^ channel activity. *The EMBO Journal*.

[62] Choe C-U, Schulze-Bahr E, Neu A (2006). C-terminal *HERG* (*LQT2*) mutations disrupt *I*
_Kr_ channel regulation 
through 14-3-3*ε*. *Human Molecular Genetics*.

[63] Allouis M, Le Bouffant F, Wilders R (2006). 14-3-3 is a regulator of the cardiac voltage-gated sodium channel Nav1.5. *Circulation Research*.

[64] Fan Y, Wang Y, Tang Z (2008). Suppression of pro-inflammatory adhesion molecules by PPAR-*δ* in human vascular endothelial cells. *Arteriosclerosis, Thrombosis, and Vascular Biology*.

[65] Cheng L, Ding G, Qin Q (2004). Cardiomyocyte-restricted peroxisome proliferator-activated receptor-*δ* deletion perturbs myocardial fatty acid oxidation and leads to cardiomyopathy. *Nature Medicine*.

[66] Frangogiannis NG, Smith CW, Entman ML (2002). The inflammatory response in myocardial infarction. *Cardiovascular Research*.

[67] Wilson PWF, D'Agostino RB, Parise H, Sullivan L, Meigs JB (2005). Metabolic syndrome as a precursor of cardiovascular disease and type 2 diabetes mellitus. *Circulation*.

[68] Miettinen H, Lehto S, Salomaa V (1998). Impact of diabetes on mortality after the first myocardial infarction. The FINMONICA Myocardial Infarction Register Study Group. *Diabetes Care*.

[69] Kostin S, Hein S, Arnon E, Scholz D, Schaper J (2000). The cytoskeleton and related proteins in the human failing heart. *Heart Failure Reviews*.

[70] Weber KT (2005). Are myocardial fibrosis and diastolic dysfunction reversible in hypertensive heart disease?. *Congestive Heart Failure*.

[71] Petrov VV, Fagard RH, Lijnen PJ (2002). Stimulation of collagen production by transforming growth factor-*β*
_1_ during differentiation of cardiac fibroblasts to myofibroblasts. *Hypertension*.

[72] Tomasek JJ, Gabbiani G, Hinz B, Chaponnier C, Brown RA (2002). Myofibroblasts and mechano-regulation of connective tissue remodelling. *Nature Reviews Molecular Cell Biology*.

[73] Eghbali M (1992). Cardiac fibroblasts: function, regulation of gene expression, and phenotypic modulation. *Basic Research in Cardiology*.

[74] Zhang H, Pi R, Li R (2007). PPAR*β*/*δ* activation inhibits angiotensin II-induced collagen type I expression in rat cardiac fibroblasts. *Archives of Biochemistry and Biophysics*.

[75] Makinde A-O, Kantor PF, Lopaschuk GD (1998). Maturation of fatty acid and carbohydrate metabolism in the newborn heart. *Molecular and Cellular Biochemistry*.

[76] Sack MN, Rader TA, Park S, Bastin J, McCune SA, Kelly DP (1996). Fatty acid oxidation enzyme gene expression is downregulated in the failing heart. *Circulation*.

[77] Planavila A, Rodríguez-Calvo R, Jové M (2005). Peroxisome proliferator-activated receptor *β*/*δ* activation inhibits hypertrophy in neonatal rat cardiomyocytes. *Cardiovascular Research*.

[78] Barish GD, Narkar VA, Evans RM (2006). PPAR*δ*: a dagger in the heart of the metabolic syndrome. *The Journal of Clinical Investigation*.

[79] Grimaldi PA (2005). Regulatory role of peroxisome proliferator-activated receptor delta (PPAR*δ*) in muscle metabolism. A new target for metabolic syndrome treatment?. *Biochimie*.

[80] Lee C-H, Olson P, Hevener A (2006). PPAR*δ* regulates glucose metabolism and insulin sensitivity. *Proceedings of the National Academy of Sciences of the United States of America*.

[81] Wallace JM, Schwarz M, Coward P (2005). Effects of peroxisome proliferator-activated receptor *α*/*δ* agonists on HDL-cholesterol in vervet monkeys. *Journal of Lipid Research*.

[82] Sprecher DL, Massien C, Pearce G (2007). Triglyceride: high-density lipoprotein cholesterol effects in healthy subjects 
administered a peroxisome proliferator activated receptor *δ* agonist. *Arteriosclerosis, Thrombosis, and Vascular Biology*.

[83] Lin H, Lin T-N, Cheung W-M (2002). Cyclooxygenase-1 and bicistronic cyclooxygenase-1/prostacyclin synthase gene transfer 
protect against ischemic cerebral infarction. *Circulation*.

